# Cement-augmented screw fixation for calcaneal fracture treatment: a biomechanical study comparing two injectable bone substitutes

**DOI:** 10.1186/s13018-020-02009-6

**Published:** 2020-11-14

**Authors:** Konrad F. Fuchs, Philipp Heilig, Miriam McDonogh, Sebastian Boelch, Uwe Gbureck, Rainer H. Meffert, Stefanie Hoelscher-Doht, Martin C. Jordan

**Affiliations:** 1 Department of Orthopaedic Trauma, Hand, Plastic and Reconstructive Surgery, University Hospital Würzburg, Oberdürrbacher Str. 6, 97080 Würzburg, Germany; 2 Department of Orthopaedic Surgery, König-Ludwig-Haus, Brettreichstraße 11, 97074 Würzburg, Germany; 3 Department for Functional Materials in Medicine and Dentistry, University of Würzburg, Pleicherwall 2, 97070 Würzburg, Germany

**Keywords:** Arthritis, Bone, Calcaneus, Cement, Fracture, Fixation, Osteoporosis, Sanders, Screw

## Abstract

**Background:**

The role of cement-augmented screw fixation for calcaneal fracture treatment remains unclear. Therefore, this study was performed to biomechanically analyze screw osteosynthesis by reinforcement with either a calcium phosphate (CP)-based or polymethylmethacrylate (PMMA)-based injectable bone cement.

**Methods:**

A calcaneal fracture (Sanders type IIA) including a central cancellous bone defect was generated in 27 synthetic bones, and the specimens were assigned to 3 groups. The first group was fixed with four screws (3.5 mm and 6.5 mm), the second group with screws and CP-based cement (Graftys® QuickSet; Graftys, Aix-en-Provence, France), and the third group with screws and PMMA-based cement (Traumacem™ V+; DePuy Synthes, Warsaw, IN, USA). Biomechanical testing was conducted to analyze peak-to-peak displacement, total displacement, and stiffness in following a standardized protocol.

**Results:**

The peak-to-peak displacement under a 200-N load was not significantly different among the groups; however, peak-to-peak displacement under a 600- and 1000-N load as well as total displacement exhibited better stability in PMMA-augmented screw osteosynthesis compared to screw fixation without augmentation. The stiffness of the construct was increased by both CP- and PMMA-based cements.

**Conclusion:**

Addition of an injectable bone cement to screw osteosynthesis is able to increase fixation strength in a biomechanical calcaneal fracture model with synthetic bones. In such cases, PMMA-based cements are more effective than CP-based cements because of their inherently higher compressive strength. However, whether this high strength is required in the clinical setting for early weight-bearing remains controversial, and the non-degradable properties of PMMA might cause difficulties during subsequent interventions in younger patients.

## Background

**Fig. 1:**
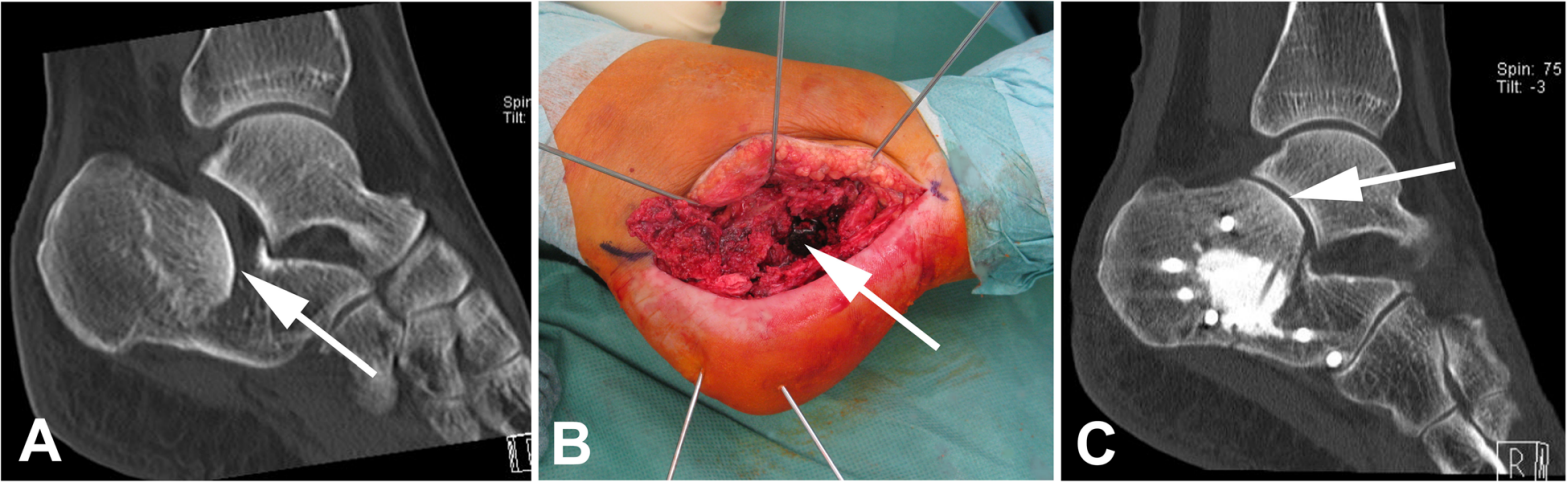
Clinical case of bone substitute used in calcaneal fracture fixation. **a** Preoperative CT demonstrating the displaced posterior joint facet (arrow) causing impaction of the cancellous bone underneath. **b** Intraoperative picture showing the osseous void (arrow) after replacement of the posterior joint surface. **c** Postoperative CT scan depicting the well-replaced posterior joint facet (arrow) and bone substitute filling up the osseous defect (plate fixation)

Calcaneal fracture treatment has been debated since the beginning of orthopedic surgery, and innumerable different surgical techniques have been described in the past; however, an ideal treatment has not yet been identified [1–3]. Regardless, most surgeons agree that open reduction by a lateral approach and plate fixation is the best treatment choice to avoid posttraumatic arthritis in patients with displaced intra-articular fractures [4–7]. Certain risks are associated with this procedure because it is invasive and not suitable for every patient or fracture. Non-union, surgical site infection, and general wound healing problems are the main reasons for poor outcomes, and comorbidities such as tobacco abuse, severe obesity, diabetes, or any form of vascular disease significantly increase the risk of developing one of these complications [8]. Therefore, surgical approaches other than open reduction and plate fixation are needed in certain cases. Minimally invasive surgical techniques can help to preserve the soft tissue and achieve anatomic reposition in patients otherwise not suitable for open reduction [9]. Percutaneous screw fixation is such a technique and has favorable outcomes; however, such fixation is sometimes difficult to apply when surgeons encounter a central osseous defect (Fig. 1). The displaced posterior articular surface of the calcaneus is usually pushed downward, compressing the cancellous bone and thereby creating a bony defect. Osseous autografts or allografts are sometimes used to fill the bony defect, strengthen the fixation, and improve healing, but their application is not a standard technique. The role of bone cement augmentation for such an indication remains unclear. We hypothesized that a combination of screw fixation and injectable bone cement yields better stability than simple screw fixation. Therefore, we conducted a biomechanical study to analyze two different injectable bone cements.

## Methods

### Specimens and fracture generation

Twenty-seven synthetic bone specimens of the right calcaneus (LD 9118; Synbone, Zizers, Switzerland) were used in this study. Previous studies have proven that the biomechanical properties are similar between synthetic bone and human specimens, and our pre-tests confirmed these findings [10]. A four-part fracture (Sanders type IIA) was generated using an oscillating saw by a technique that has been proven reproducible in other studies [11]. To mimic a central bony defect, a reamer was used to create a predefined defect in the subtalar region. All specimens were then checked for equal defect and fragment sizes and randomly separated to three groups (Table 1).

**Table 1:** Assessment of three study groups

Group	Osteosynthesis	Bone cement
**A**	2 × 3.5-mm cortical screw and 2 × 6.5-mm cancellous screw	None
**B**	2 × 3.5-mm cortical screw and 2 × 6.5-mm cancellous screw	Graftys® QuickSet; calcium-deficient hydroxyapatite, slow biodegradability
**C**	2 × 3.5-mm cortical screw and 2 × 6.5-mm cancellous screw	Traumacem™ V+, 45% polymethylmethacrylate +40% zirconium dioxide +15% hydroxyapatite, no biodegradability

### Experimental groups

*Group A* functioned as the control group. The fracture was reduced and then fixed with four screws. Two 6.5-mm fully threaded cancellous screws were advanced in the posterior-anterior direction. The starting point was the calcaneal tubercle (Cancellous Bone Screw, Reference 418.075, length of 75 mm; DePuy Synthes, Warsaw, IN, USA). A 3.2-mm drill bit and a 6.5-mm tap were used. Next, two 3.5-mm fully threaded cortical screws were used in the lateral-to-medial direction in the subtalar region (References 406.030 and 406.035, lengths of 30 mm and 35 mm; DePuy Synthes). A 2.5-mm drill bit and a 3.5-mm tap were used.

The calcaneal fracture in *Group B* was similarly reduced and fixed with four screws, and the osseous defect was filled using 5 ml of injectable CP-based cement (Graftys® QuickSet, Reference GYQSMV1Q8; Graftys, Aix-en-Provence, France). Graftys® consists of a slowly degradable hydroxyapatite matrix. The specimens were left to set for 48 h in an incubator at 37 °C with an adequate humidity.

**Fig. 2:**
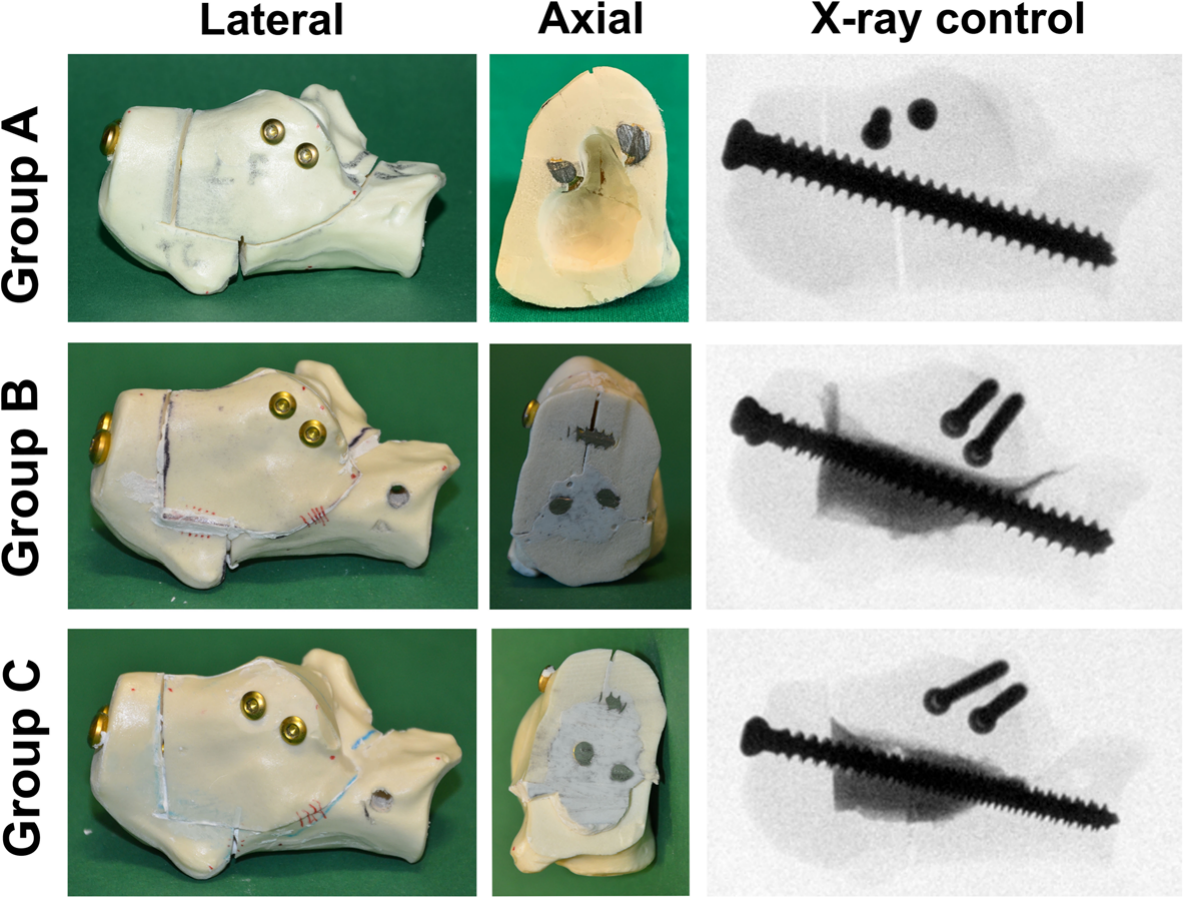
Study groups. **a** Control group with screws only. **b** Screw osteosynthesis and calcium phosphate-based bone substitute. **c** Screw osteosynthesis and polymethylmethacrylate-based bone substitute. All specimens were checked under intensifier control for similar implant placement

The calcaneal fracture in *Group C* was reduced and fixed with four screws in the abovementioned manner. The bone defect was filled using 5 ml of injectable PMMA-based bone cement (Traumacem™ V+, Reference 07.702.040S; DePuy Synthes). Traumacem™ V+ is a composite of acrylic bone cement in conjunction with ceramics consisting of 45% PMMA, 40% zirconium dioxide (radiopaque), 14.5% hydroxyapatite, and 0.5% benzoyl peroxide. The specimens were left to set for 48 h in an incubator at 37 °C with an adequate room humidity. All specimens were photographed and checked under an X-ray image intensifier for similar screw placement and cement localization (Fig. 2).

### Biomechanical testing

**Fig. 3:**
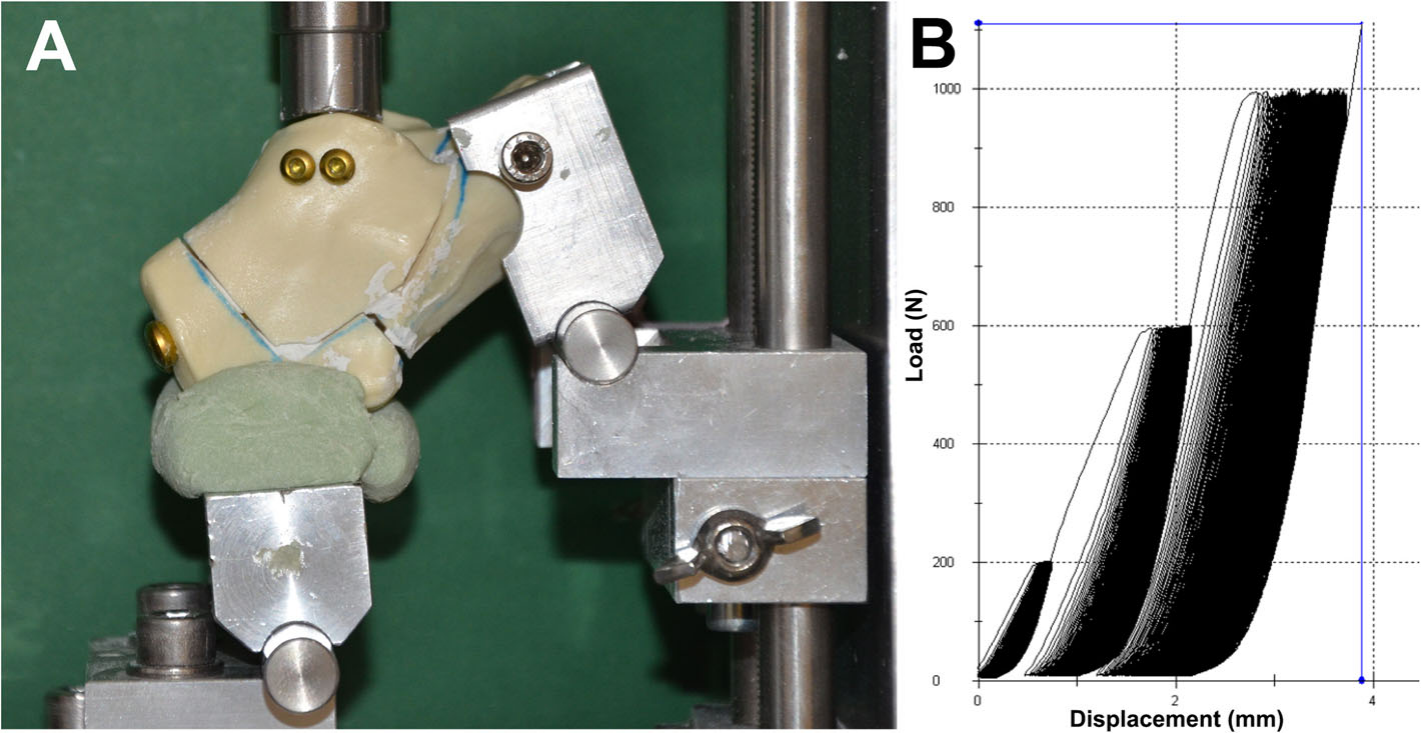
Test setup. **a** Fixation device for calcaneus with axial load application in the material testing machine. **b** Load–displacement curve under different load levels

All specimens were installed in a custom-made device that was established in a previous study [12]. The specimens were fixed anteriorly with two steel pins, which allowed rotation in the calcaneocuboid joint. The plantar calcaneal tuberosity was embedded in bone cement (PMMA; Heraeus, Hanau, Germany). Testing was conducted using a material-testing machine (Zwick/Roell Z020; Zwick GmbH & Co., Ulm, Germany) and the appropriate software (testXpert version 3.6; Zwick/Roell). We applied an axial force to the posterior articular facet using a custom-made indenter mimicking the talus (Fig. 3). The indenter was attached to a 20-kN load cell. Our test protocol consisted of cyclic testing at three different load levels and a final load test up to 2000 N. Ten primary settling cycles from 0 to 10 N were performed with a displacement speed of 50 mm/min. Next, 500 cycles were performed at 200, 600, and 1000 N. The protocol ended with a load-to-failure test. The loads are analogous to partial and full weight-bearing and have thus been used in other studies [13]. No relevant changes were observed beyond 1500 test cycles. That is why we choose this number for cyclic loading. We measured the peak-to-peak displacement (PTPD; mm) at 200, 600, and 1000 N; maximum displacement at 2000 N (mm); and stiffness (N/mm). The PTPD was measured as the distance of the first peak load to the last of the respective load cycle. Total displacement was measured by the distance from the beginning until the final load of 2000 N. The stiffness was determined using the gradient of the linear part of the load–displacement curve generated by the software.

### Statistical analysis

Microsoft Excel 2010 (Microsoft Corp., Redmond, WA, USA) was used for data collection. The data were analyzed using SPSS Statistics 25 (IBM Corp., Armonk, NY, USA). A power analysis was performed in previous test using a power of 80% and a significance level of 5%, which showed that the sample size was adequate (*N* = 9). The results are presented as mean with standard deviation. All data were statistically analyzed for a normal distribution using the Shapiro–Wilk test. Data with a normal distribution were compared using analysis of variance and the Bonferroni test. A *p* value of < 0.05 was considered statistically significant. Data without a normal distribution were analyzed using the Kruskal–Wallis test.

## Results

### PTPD at 200, 600, and 1000 N

**Fig. 4:**
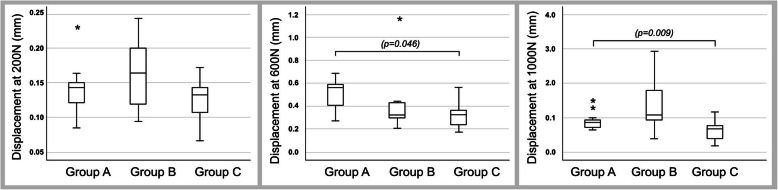
Results of peak-to-peak displacement under 200-, 600-, and 1000-N loads. There was significantly less displacement of the posterior articular surface in specimens fixed with screws and a polymethylmethacrylate-based bone substitute under high load. *Outlier

The mean PTPD under the 200-N load measured by the software was 0.14 ± 0.14 mm in group A, 0.16 ± 0.2 mm in group B, and 0.12 ± 0.01 mm in group C. The data were normally distributed. There was no statistically significant difference among the groups (*p* = 0.168). The mean PTPD under the 600-N load was 0.5 ± 0.04 mm in group A, 0.51 ± 0.13 mm in group B, and 0.32 ± 0.04 mm in group C. The data were not normally distributed. There was a significant difference between groups A and C (*p* = 0.046), but no other significant differences. The mean PTPD under the 1000-N load was 0.92 ± 0.1 mm in group A, 1.45 ± 0.31 mm in group B, and 0.63 ± 0.1 mm in group C. The data were not normally distributed. There was a significant difference between groups A and C (*p* = 0.038), but no other significant differences (Fig. 4).

### Maximum displacement at 2000 N

**Fig. 5:**
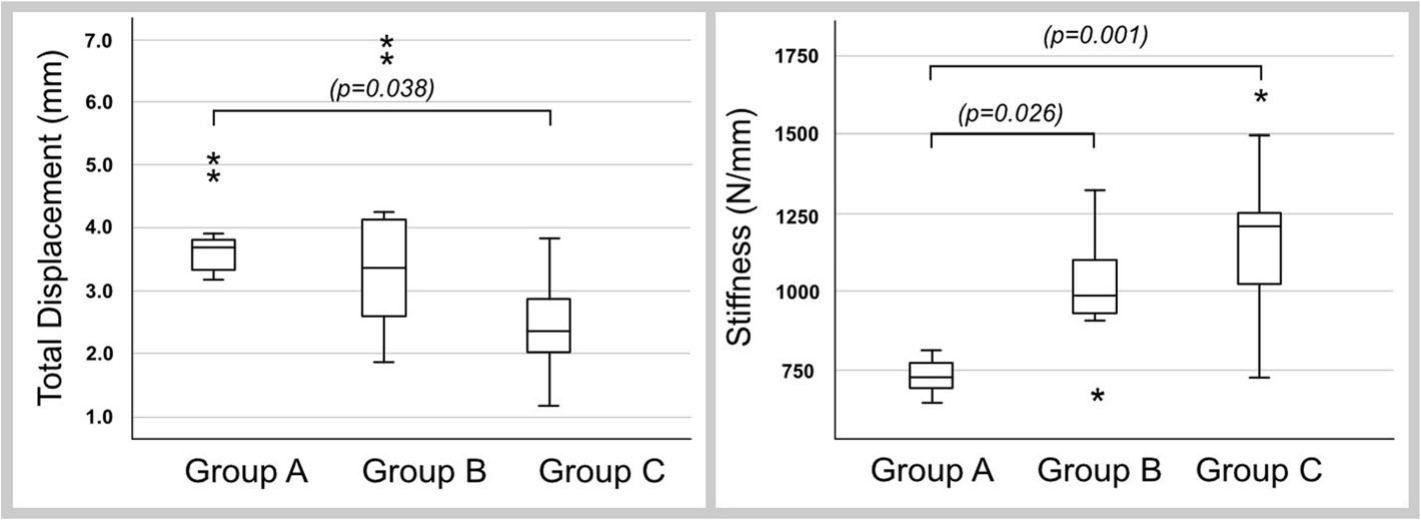
Maximum displacement confirmed findings of peak-to-peak displacement. Stiffness shows that calcium phosphate bone substitute is also beneficial

The data for maximum displacement were not normally distributed. The mean maximum displacement at 2000 N was 3.83 ± 0.22 mm in group A, 3.79 ± 0.63 mm in group B, and 2.45 ± 0.26 mm in group C. A significant difference was found between groups A and C (*p* = 0.009) (Fig. 5).

### Stiffness

The data for stiffness showed a normal distribution. The mean stiffness was 730 ± 18 N/mm in group A, 1006 ± 60 N/mm in group B, and 1200 ± 100 N/mm in group C. There was a significant difference between groups A and B (*p* = 0.026) and between groups A and C (*p* = 0.001).

## Discussion

This study was performed to clarify the biomechanical value of injectable bone cement added to screw fixation for fractured calcanei. With respect to displacement and stiffness, the addition of bone cement produced higher stability, especially in loads of > 200 N. Bone cement consisting partially of PMMA yields better stability than bone cement based on CP.

It is important to know that bone substitutes are available for many years by now and many different studies have analyzed its use in calcaneal fracture treatment; however, its use is not well established or accepted for calcaneal fracture care among most surgeons.

Traumacem™ V+ and Graftys® are both approved bone substitutes to fill bone defects or augment osteosynthesis. While similar in application, they have profound differences. Traumacem™ V+ consists mainly of polymerized acrylic resin (PMMA), produced from a mix of methyl methacrylate monomer, and pre-polymerized PMMA solid in an exothermic reaction [14]. During the setting, temperatures rise above 60 °C, yielding an inert, non-biodegradable block. CP cements were developed as a needed degradable alternative to PMMA-based substitutes. Self-setting CP cements form an osteoconductive, degradable bone cement by a dissolution-reprecipitation process [15]. The Graftys® used in this study sets under a pH of > 4.2 and produces a slowly degrading hydroxyapatite, mimicking the composition and crystallinity of the inorganic phase of the bone matrix [10]. The mean compressive strength of Graftys® was 19.0 ± 2.5 MPa in our previous tests (manufacturer information: 24 MPa after 24 h) [10]. The compressive strength of Traumacem™ V+ is approximately 90 MPa, which is comparable with that of pure PMMA (approximately 100 MPa) [14]. Mixing PMMA with hydroxyapatite improves its handling properties [16]. The cancellous calcaneus bone has a compression strength of around 3.22 MPa [17].

Previous findings in biomechanical studies regarding bone substitutes in calcaneal fracture treatment were promising. No matter if bone substitutes were applied by injection through cannulated screws or as solid block, the stability always increased [11, 13, 18, 19]. The calcanei treated with bone cement showed significantly less deformation in cyclic testing, and the authors reported a large increase in strength of the construct that possibly leads to more rapid rehabilitation. Our findings raise concerns regarding the utility of CP-based cements for this indication. Most biomechanical parameters were not significantly changed by CP-based cement application in our model.

The use of CP-based cements was further assessed in clinical studies. Ditzen and Börner treated 18 cases of calcaneal fractures with indirect reduction, K-wire fixation, and CP cement augmentation (Norian SRS applied through the fracture line) and reported good results [20]. Wee and Wong evaluated 10 patients treated with percutaneous K-wire fixation and CP-based cement augmentation (Norian SRS). Outcome measures such as the visual analog scale score, Maryland Foot Score, and Short Form 36 score revealed promising results [21]. Elsner et al. followed-up 18 patients treated with open reduction, internal fixation, and augmentation with CP-based cement. Biopsies were taken at the time of hardware removal. The authors found that patients treated with bone substitute could bear weight earlier. Interestingly, complete resorption of the bone cement and remodeling had not occurred at the final check-up 3 years after surgery [22]. Schildhauer et al. treated 36 joint depression type calcaneal fractures with standard open reduction, plating, and calcium phosphate cement augmentation (Norian SRS). Compared with the abovementioned percutaneous techniques, the open reduction performed in this study had a high wound infection rate of 11% [23]. Thordarson and Bollinger used open reduction and plate osteosynthesis in combination with calcium phosphate cement (Norian SRS) to obtain anatomical reduction in fractured calcanei. They followed-up nine patients; none of whom had a loss of reduction. Degradation of the bone cement was not observed in computed tomography scans after 12 months [24].

All the abovementioned clinical studies confirm the interest in bone substitutes for treatment of calcaneus fractures. Although several new bone cements were introduced since the year 2000, the data gathered to date have not yet changed the daily practice. Overall concerns like cement leakage, inflammation, foreign body reaction, or general precaution prevent widespread clinical use of bone substitutes in calcaneal fracture care. Our study can add biomechanical knowledge but clinical concerns persist.

## Data Availability

The datasets analyzed during the current study are available from the first author on reasonable request.

## Abbreviations

AOFAS: American Orthopaedic Foot and Ankle Society
PMMA: Polymethylmethacrylate
CP: Calcium phosphate
PTPD: Peak-to-peak displacement
